# The Characteristics and Expression of *RBX1* Gene Involved in Ovarian Development of *Scylla paramamosain*

**DOI:** 10.3390/ijms27010363

**Published:** 2025-12-29

**Authors:** Fengying Zhang, Ting Huang, Yuanhao Ren, Ming Zhao, Wei Wang, Zhiqiang Liu, Keyi Ma, Yin Fu, Wei Chen, Lingbo Ma, Chunyan Ma

**Affiliations:** 1 Key Laboratory of East China Sea Fishery Resources Exploitation, Ministry of Agriculture and Rural Affairs, East China Sea Fisheries Research Institute, Chinese Academy of Fishery Sciences, Shanghai 200090, China; 2 College of Fisheries and Life Science, Shanghai Ocean University, Shanghai 201306, China

**Keywords:** *Scylla paramamosain*, *RBX1*, oocyte maturation, ovary, hepatopancreas, development

## Abstract

Ring Box Protein-1 (RBX1) is an essential component of the Skp1-cullin-F-box protein (SCF) E3 ubiquitin ligase, which is involved in the regulation of oocyte maturation in the form of ubiquitination substrate modification. In this study, a sequence of RBX1 (*Sp-RBX1*) was identified and analyzed using bioinformatics methods from the transcriptome data of *Scylla paramamosain*. The length of *Sp-RBX1* cDNA sequence was 1247 bp, consisting of a 336 bp open reading frame (ORF). Sequence analysis revealed that the protein contained a C-terminal modified RING-H2 finger domain, with two zinc binding sites and a Cullin binding site, classifying it as a member of the RBX1 superfamily. The results of real-time fluorescence quantitative PCR (RT-qPCR) showed that *Sp-RBX1* expression in the ovary was low at stages I and II, then significantly increased from stage III to V (*p* < 0.05), which indicated that it might be closely related to the maturation of oocytes. It also peaked at stage II in the hepatopancreas, then sharply declined from stages III to V. The expression pattern might be related to the accumulation of fat in the early development of hepatopancreas. Furthermore, we characterized the expression of *Sp-RBX1* induced by follicle-stimulating hormone (FSH) and estradiol (E2) hormones. The results showed that the expression in the ovary was up-regulated by FSH and significantly inhibited by E2. The expression in the hepatopancreas increased only at 0.5 µmol/L concentration of FSH, and decreased in other groups. Conversely, it was up-regulated by E2. Thus, the expression of *Sp-RBX1* was influenced by FSH in a concentration-dependent manner. These findings could offer valuable insights for further research on ovarian maturation in crustaceans.

## 1. Introduction

The mud crab *Scylla paramamosain* (Crustacea: Decapoda: Portunidae: *Scylla*) is an economically important species in China and Southeast Asia [1]. In China, the total yield of *S. paramamosain* amounted to 224 thousand tons in 2023 [2]. Female crabs possess more economical and nutritional value, due to their matured ovaries, than males. The development of ovaries can be divided into five stages: undeveloped (stage I), pre-vitellogenic (stage II), early vitellogenic (stage III), late vitellogenic (stage IV), and mature (stage V) [3]. Oocyte maturation is a complex process involving the interaction of extra-ovarian and intra-ovarian signals [4,5]. The maturation process can be affected by some hormones, such as follicle-stimulating hormone (FSH) and estradiol (E2) [6,7,8,9].

Ring Box Protein-1 (RBX1) is an important component involved in ubiquitination processes [10]. The process targets the proteasome-mediated degradation of multiple proteins [11]. RBX1 protein is involved in various cellular processes by targeting multiple substrates, such as cyclin regulation, cell transcription, and signal transduction [12,13]. RBX1 protein is expressed in reproductive cells, and it is potentially significant as a maternal protein in the maturation of oocytes, early embryonic development, and other related processes [14]. It has been reported that reproductive cells exhibited evident abnormalities in meiosis and cell proliferation after RNAi-mediated knockdown of *RBX1* gene [15]. RBX protein homologs also influence the development of reproductive cells [16], and the absence of *RBX1* gene can result in individual mortality and male infertility [17].

So far, the research on RBX1 protein in the development of aquatic organisms is limited. RBX1 plays a key role in spermatogenesis of *Eriocheir sinensis* through forming a complex with Cullin4 [18]. RBX1 is essential for cardiac wall morphogenesis in zebrafish [19]. RBX1 protein exhibits ubiquitin ligase activity and indirectly contributes to the immune response in *Haliotis diversicolor supertexta* through self-ubiquitination [20]. In this study, we focused on the relationship between the *RBX1* gene and oocyte maturation. We obtained and analyzed the cDNA sequence of RBX1 in *S. paramamosain*, examined RBX1 expression patterns in various tissues and during different ovarian stages, and subsequently characterized the hormone-induced expression of *Sp-RBX1* gene by FSH and E2 in vitro. This research provides a theoretical reference for further study of RBX1 on oocyte maturation and ovarian development in *S. paramamosain*.

## 2. Results

### 2.1. RBX1 cDNA Sequence and Coded Protein

**Figure 1:**
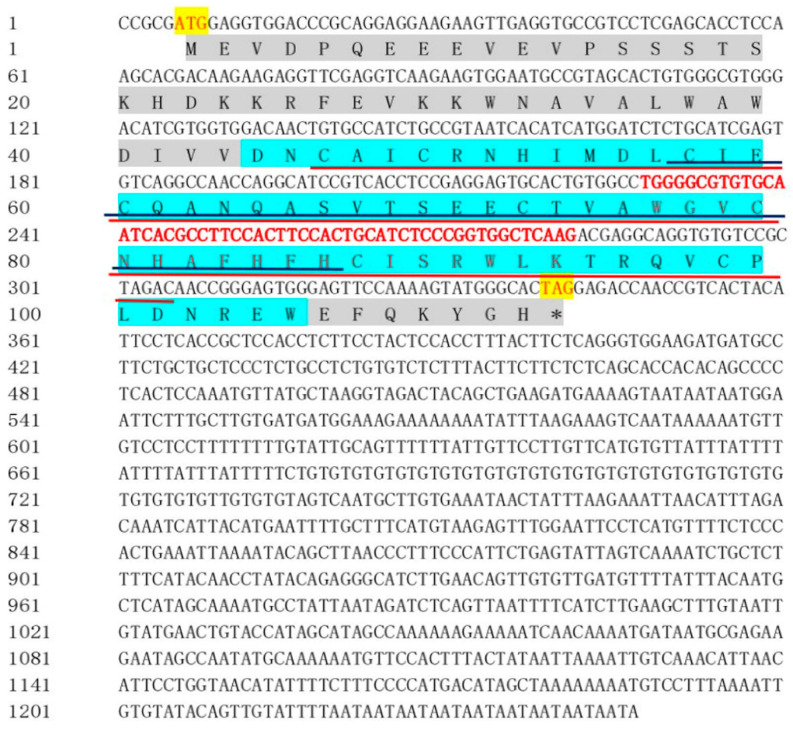
cDNA and deduced amino acid sequence of *RBX1* from *S. paramamosain*. Note: The start codon (ATG) and stop codon (TAG) are in the yellow box; the asterisk "*" is marked as a termination codon; the gray regions represent the C-terminal conserved domain: the blue shadow region indicates RING finger domain (132~317); the red line indicates a Zn binding site (138–305), the dark blue line indicates another zinc binding site (171–260), and the red sequence indicates a Cullin binding site (228–281).

In this study, a homolog of RBX1 was obtained from our transcriptome data and verified to be the RBX1 ortholog, which was named Sp-RBX1 and deposited in GenBank (Accession No. PX688505). The cDNA was 1247 bp in length, with a 336 bp ORF, a 5 bp 5′-untranslated region (5′ UTR), and a 906 bp 3′-untranslated region (3′ UTR). This ORF encoded a 111-amino-acid protein, and the calculated molecular mass was 13.08 kDa (Figure 1). The possible molecular formula of the RBX1 protein was C_573_H_867_N_163_O_170_S_10_, and the isoelectric point (pI) was 5.63. The predicted results indicated a total of 17 negatively charged amino acids, and 12 positively charged amino acids. The proportion of valine and glutamic acid in the amino acid composition was up to 9.8%, followed by alanine (8.0%). The RBX1 protein was predicted to contain a modified RING finger domain at its C-terminus, spanning amino acid residues 132 to 317. This domain included two zinc binding sites (138–305 and 171–260) and a Cullin binding site (228–281).

### 2.2. RBX1 Multiple Sequence Alignment and Phylogenetic Analysis

**Figure 2:**
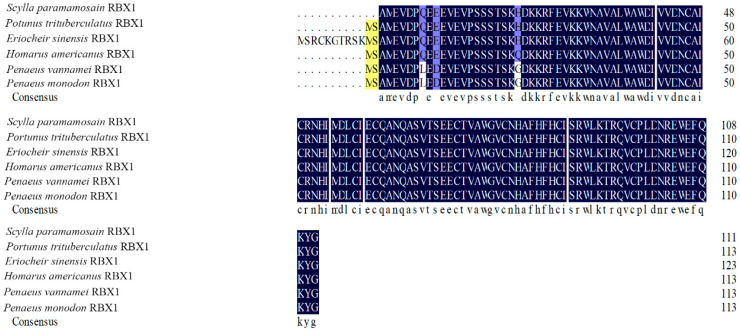
Alignment of the deduced amino acid sequence of Sp-RBX1 protein with other species. Note: The shadowed regions represent homology of amino acids: black: similarity = 100%, yellow: similarity > 75%, blue: similarity > 33%.

**Figure 3:**
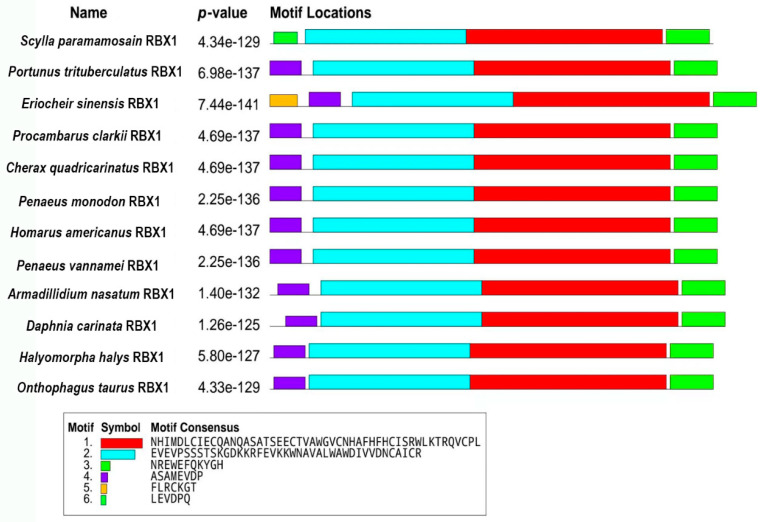
Comparison of conserved motifs between Sp-RBX1 protein and other species. Note: motif 1 (red), motif 2 (blue), and motif 3 (light green) were conserved motifs of RBX1; motif 4 (purple), motif 5 (orange), and motif 6 (dark green) were typical conserved elements.

**Figure 4:**
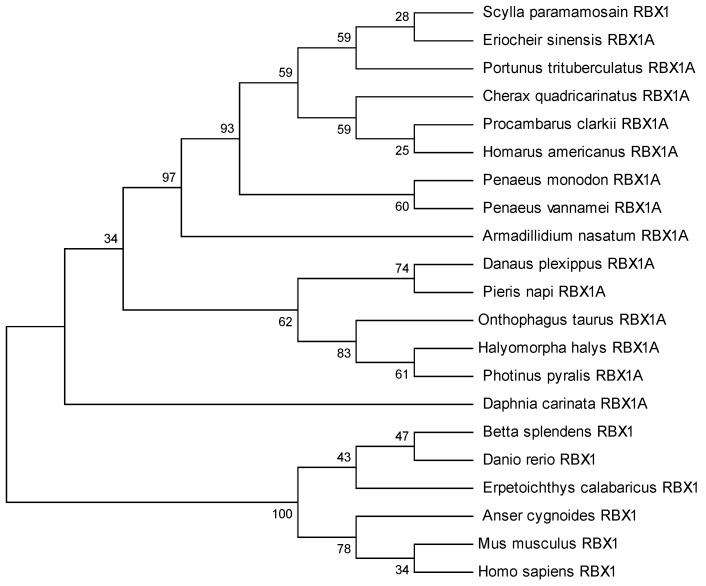
The phylogenetic tree of RBX1 amino acid sequences from multiple species according to neighbor-joining method.

The amino acid sequence comparison of RBX1 revealed the highest homology with *Portunus trituberculatus* and *E. sinensis* (100%), followed by *Homarus americanus* (99.11%), *Penaeus vannamei* (97.32%), and *P. monodon* (97.30%) (Figure 2). There were three conserved motifs (motif 1, motif 2, and motif 3) and three typical conserved elements (motif 4, motif 5, and motif 6) in RBX1 protein, among twelve species, in which motif 1 and motif 2 were RING finger domains (Figure 3). The phylogenetic tree analysis showed that *S. paramamosain* is most closely genetically related to *P. trituberculatus* and *E. sinensis*. The crustaceans clustered together on one branch, and insects generally formed another branch, while fish and mammals were grouped together on a separate branch (Figure 4).

### 2.3. Analysis of Expression Pattern of RBX1 Gene In Vivo

**Figure 5:**
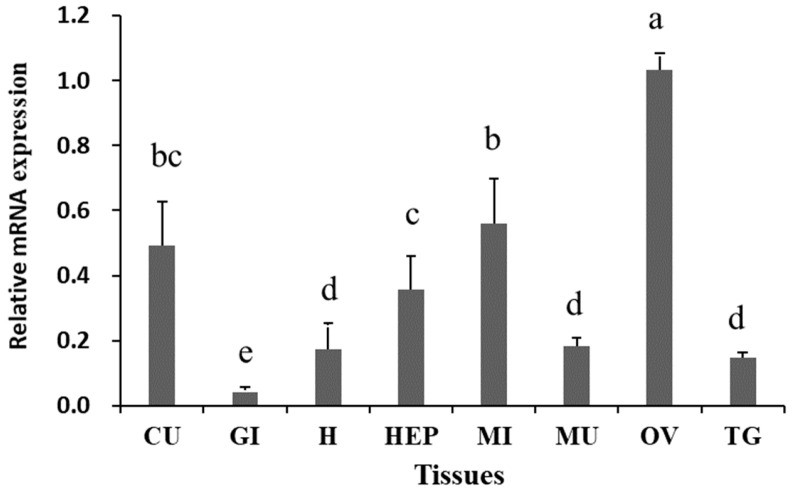
Expression analysis of *Sp-RBX1* in different tissues. Note: CU: cuticle; H: heart; MI: intestine; MU: muscle; HEP: hepatopancreas; GI: gill; TG: thoracic ganglion; OV: ovary. Different letters on the graph indicate significant differences (*p* < 0.05).

**Figure 6:**
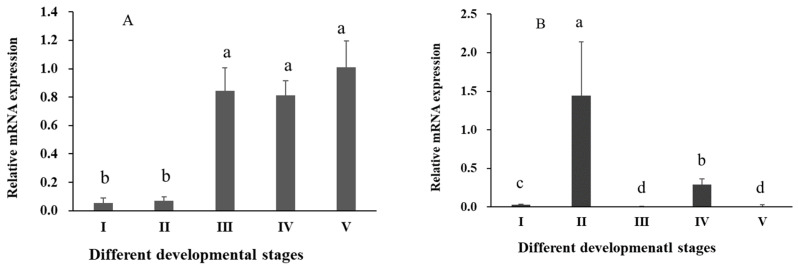
Expression of *Sp-RBX1* during different development stages in ovaries and hepatopancreas. Note: (**A**), expression in ovary, I–V: five stages of ovarian development; (**B**), expression in hepatopancreas, I–V: five stages of hepatopancreatic development. Different letters on the graph indicate significant differences (*p* < 0.05).

The results of RT-qPCR revealed significant differential expression of *Sp-RBX1* in eight tissues, with the highest expression observed in the ovary, followed by intestine, cuticle, and hepatopancreas, while the lowest expression level was detected in the gill (*p* < 0.05) (Figure 5). During the ovarian development of *S. paramamosain*, the expression of *Sp-RBX1* in the ovary was relatively low in stages I and II, and significantly increased from stage III to V in the ovary (*p* < 0.05) (Figure 6A). The expression of *Sp-RBX1* in the hepatopancreas peaked at stage II, but dropped significantly from stage III onward, remaining low through stage V (*p* < 0.05) (Figure 6B).

### 2.4. Analysis of Effects of FSH and E2 on Expression Patterns of Sp-RBX1 In Vitro

**Figure 7:**
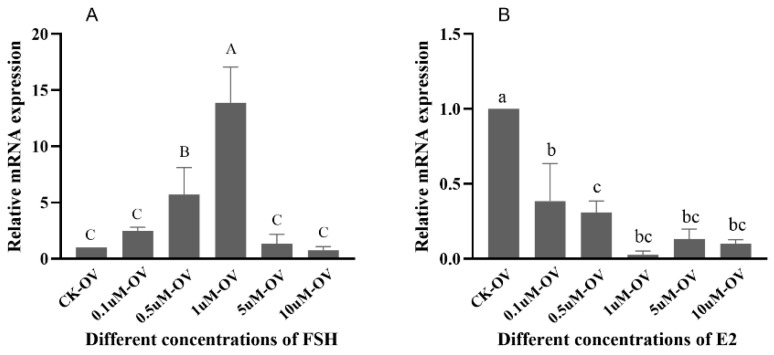
The effects of different FSH and E2 concentrations on *Sp-RBX1* in ovaries in vitro. Note: (**A**), the effect of FSH; (**B**), the effect of E2. Different case letters indicate significant differences; the capitals indicate FSH, and lowercases indicate E2 (*p* < 0.05).

The expressions of *Sp-RBX1* in the ovarian tissue increased gradually with FSH concentrations from 0.1 to 1 µM, reaching its peak at 1 µM. However, its expression sharply decreased at concentrations of 5 and 10 µM (*p* < 0.05) (Figure 7A). Compared to the control group, the expressions of *Sp-RBX1* were significantly inhibited under E2 concentrations ranging from 0.1 to 10 µM (*p* < 0.05) (Figure 7B).

**Figure 8:**
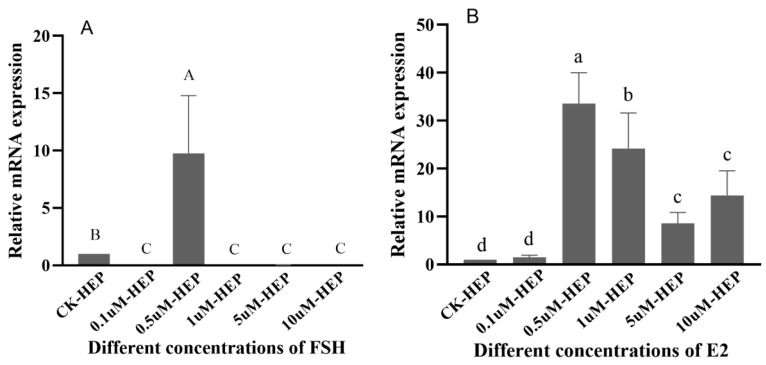
The effects of different FSH and E2 concentrations on *Sp-RBX1* in hepatopancreas in vitro. Note: (**A**), the effect of FSH; (**B**), the effect of E2. Different case letters indicate significant differences; the capitals indicate FSH, and lowercases indicate E2 (*p* < 0.05).

The regulatory effect of FSH on *Sp-RBX1* in the hepatopancreatic tissue was up-regulated at the concentration of 0.5 µM. However, other concentrations of FSH had an inhibitory effect on the expression of *Sp-RBX1* (*p* < 0.05) (Figure 8A). *Sp-RBX1* expression showed a notable up-regulation under the incubation of E2, with the concentrations ranging from 0.5 to 10 µM (*p* < 0.05), reaching the highest level at the concentration of 0.5 µM (*p* < 0.05) (Figure 8B).

## 3. Discussion

RBX1 homologous proteins are evolutionarily conserved from plants to mammals [21]. In this study, we obtained the cDNA sequence of RBX1 from the mud crab. The predicted Sp-RBX1 protein contains a C-terminal modified RING-H2 finger domain, crucial for its ligase activity [22]. This domain includes two zinc binding sites and a Cullin binding site, similar to most RBX1 superfamily members [23]. This conserved domain is homologous to RBX protein families, which indicates that Sp-RBX1 is a member of the RBX superfamily. The evolutionary tree of RBX1 proteins indicated a high evolutionary homology between *S. paramamosain* and other crabs, which was consistent with previous reports [24]. The evolutionary tree analysis of RBX1 conformed to the biological classification and evolutionary characteristics.

The function of a gene is closely related to specific tissue distribution [25]. In this study, it was found that *Sp-RBX1* was expressed in multiple tissues of the mud crab, with the highest level observed in the ovary, which was similar to the expression pattern in the gonad of *E. sinensis* [18]. Thus, we supposed that *RBX1* might potentially be involved in regulating the growth, development, and reproductive processes of crustaceans, particularly in gonadal development. However, further study is required to determine the specific underlying mechanisms.

RBX1 protein plays a key role in oogenesis and ovarian maturation [26]. In ovaries, the expression of *Sp-RBX1* gene was low in stage I and stage II, and significantly increased from stage III to stage V. The elevated expression of *RBX1* during the middle and late stages of ovarian development might be due to its participation in the ovarian development process [27]. During ovarian development, the hepatopancreas synthesizes and accumulates lipids, then transports them into the ovary to provide the energy and material basis for vitellogenesis and oocyte maturation [28]. In the hepatopancreas of *S. paramamosain*, the level of total lipid at stage II was significantly greater than those at stages III, IV, and V [29]. The highest expression of *Sp-RBX1* in the hepatopancreas at stage II might be correlated with the high energy requirements of subsequent ovum formation and fat accumulation in the ovary.

FSH stimulates follicle growth and oocyte maturation, playing a critical role in reproductive physiology. In this study, FSH up-regulated *Sp-RBX1* expression in a concentration-dependent manner, within the range of 0.1 µM to 1 µM, and the most significant effect was observed at 1 µM in ovaries. However, higher FSH concentrations suppressed *Sp-RBX1* expression, suggesting a biphasic regulatory role. This indicated that RBX1 might be involved in oocyte maturation through FSH-mediated hormonal regulation, as supported by previous studies [30,31]. Beyond its role in reproductive regulation, FSH has been shown to influence lipid metabolism [32]. The expression of *Sp-RBX1* was obviously up-regulated under the concentration of 0.5 µM of FSH. This function might be mediated through its interaction with *Sp-RBX1* in the hepatopancreas, which served as the primary lipid storage organ in *S. paramamosain*. These findings suggest that FSH, along with other reproductive hormones, might play a dual role in both reproductive and metabolic regulation in crustaceans.

E2 is an important sex steroid hormone involved in regulation of animal lipid metabolism [33]. The expression of *Sp-RBX1* was up-regulated under the different concentrations of E2, ranging from 0.5 to 10µM, in the hepatopancreas. It was obviously up-regulated under the concentration of 0.5 µM of E2, which suggested the concentration of 0.5 µM might be the optimal concentration to exert biological effects. These findings indicated that hormonal regulation influenced the expression of *Sp-RBX1* and might participate in the fat metabolism processes of *S. paramamosain*. In previous studies, the positive effects of E2 on ovarian development were not detected in some crustaceans at a particular ovarian stage [34,35]. The expression of *Sp-RBX1* was low under E2 treatment in ovarian tissue. The possible reason is that E2 might promote oocyte maturation through nutrient provision to the ovaries, mediated by the hepatopancreas, rather than acting directly on ovarian cells.

In conclusion, the cDNA sequence of *Sp-RBX1* was obtained, and its sequence characteristics and expression patterns in different tissues and different stages of ovarian development were analyzed. We proposed a preliminary speculation on the regulatory mechanism of *Sp-RBX1* in the ovarian development and maturation of *S. paramamosain*. In addition, through culture experiments in vitro, we demonstrated that *Sp-RBX1* might be regulated by FSH and E2 to influence oocyte development and ovarian maturation in *S. paramamosain*. These findings could offer valuable insights for further research on ovarian development in *S. paramamosain.*

## 4. Materials and Methods

### 4.1. Ethics Statement

All animal experiments in this study were conducted in accordance with the relevant national and international guidelines. Our project was approved by the East China Sea Fisheries Research Institute, Chinese Academy of Fishery Sciences, 20250410-1, on 10 April 2025. Our study did not involve endangered or protected species.

### 4.2. Collection of Experimental Samples

The healthy female mud crabs were selected from the Zhejiang Ninghai Research Center, East China Sea Fisheries Research Institute, Chinese Academy of Fishery Sciences. Ovarian and hepatopancreatic tissues from stage I to stage V (3 individuals per stage) were collected to analyze the expression patterns. In addition, 6 tissues, including thoracic ganglion, epidermis, heart, intestine, gills, and muscles, from adult crabs at stage III were sampled (3 replicates) to analyze tissue-specific expression. Each tissue was placed in RNA keeper tissue stabilizer and stored at −70 °C.

### 4.3. RNA Extraction and cDNA Synthesis

Total RNA was extracted using UNIQ-10 column Trizol extraction kit (TransGen Biotech Co., Ltd., Beijing, China) according to the manufacturer’s instructions. The concentration and quality of RNA were determined using a NanoOne spectrophotometer (Thermo Scientific, Shanghai, China). First-strand cDNA was synthesized using *EasyScript* One-Step gDNA Removal and cDNA Synthesis SuperMix reverse transcription kit (TransGen Biotech Co., Ltd., Beijing, China) according to the manufacturer’s protocol, and then stored at 4 °C.

### 4.4. Validation of RBX1 Sequence

The cDNA sequence of RBX1, designated *Sp-RBX1*, was identified from the full-length transcriptome library of *S. paramamosain* in our laboratory. The primers for sequence verification were designed by the software Primer Premier 5.0 (Table 1). The PCR reaction was performed in a 25 μL volume consisting of 2× Power Taq PCR MasterMix, 1 μL of each primer (10 μM), 1 μL of template cDNA, and ddH_2_O. The PCR reactions were carried out under the following conditions: 5 min pre-denaturation at 94 °C; 42 cycles of 30 s at 94 °C, 45 s at 52 °C, and 45 s at 72 °C; and a final extension at 72 °C for 7 min. The PCR amplification products were checked with 1% agarose gel electrophoresis and sent to Shanghai Jieli Biological Limited Company (Shanghai, China) for sequencing.

### 4.5. Bioinformatics Analysis of Sp-RBX1 Sequence

The open reading frame (ORF) of the *Sp-RBX1* sequences was identified using NCBI (https://www.ncbi.nlm.nih.gov/orffinder/ accessed on 29 December 2024). The ExPASy portal (https://www.expasy.org/ accessed on 29 December 2024) was used to predict the protein molecular weight, molecular structure, and isoelectric point. The homologous RBX1 amino acid sequences were downloaded from NCBI (https://www.ncbi.nlm.nih.gov/ accessed on 29 December 2024). These species and accession numbers were listed in Table 2. The multiple sequence comparison analysis was carried out by DNAMAN V6 software to determine the similarity of RBX1 amino acid sequences among different species. A phylogenetic tree was constructed, using the above sequences, with the maximum likelihood method (ML) in the MEGA 11 software. The bootstrap value was set to 1000, and the Jones–Taylor–Tornton (JTT) model was used.

### 4.6. Expression Profile of RBX1 in Various Tissues and Developmental Stages

Real-time fluorescence quantitative PCR (RT-qPCR) was performed to determine the relative expression levels of Sp-RBX1 in different tissues, and its temporal expression profile across various stages of ovarian and hepatopancreatic development. Primers for the RT-qPCR reaction of Sp-RBX1 were designed using Primer Premier 5.0. The 18S rRNA was used as the internal reference gene [36] (Table 1). The RT-qPCR reaction was performed in a total volume of 20 μL, consisting of 2 μL of template cDNA, 10 μL of SYBR Primix Ex Taq, 0.8 μL of each primer (10 μM), and 6.4 μL of ddH_2_O. The RT-qPCR reactions were performed under the following conditions: 30 s initial denaturation at 94 °C; 40 cycles of 5 s at 94 °C, and 30 s at 60 °C. The relative expression of Sp-RBX1 in the different samples was calculated using the 2^−∆∆Ct^ method.

### 4.7. Analysis of Expression Patterns of Sp-RBX1 Regulated by FSH and E2

FSH was completely dissolved in 10 mM PBS (pH 7.4) containing 1% bovine serum albumin (BSA), then mixed and diluted to the working solutions, with final concentrations of 10 µM, 50 µM, 100 µM, 500 µM, and 1000 µM. The E2 hormone was completely dissolved in 1% DMSO and diluted to the same final concentrations as FSH. Tissues, including ovarian and hepatopancreatic tissues, were collected from 3 healthy female mud crabs (stage III). The tissues were cut into 25 mg pieces with sterilized scissors, rinsed with sterile normal saline 4 times, and placed into sterile 24-well Petri dishes (Corning, New York, NY, USA). They were then cultured on a shaker at 25 °C. A 20 µL hormone working solution was added to 1980 µL of DMEM high-glucose medium (Thermo Fisher Scientific, Waltham, MA, USA) to achieve final concentrations of 0.1 µM, 0.5 µM, 1 µM, 5 µM, and 10 µM for each hormone. The control group was treated with 100 µM of PBS for FSH group and 0.01% of DMSO for E2 group. After 6 h of culture on the shaker, the tissues were stored in −80 °C refrigerator until RT-qPCR. All experiments were independently repeated three times.

### 4.8. Data Analysis of RT-qPCR

As for the results of RT-qPCR, all data were calculated to derive the mean and standard error (SE). Homogeneity of variances was performed by Levene’s test, followed by one-way analysis of variance (ANOVA) with Scheffé’s post hoc analysis. Differences were considered significant at *p* < 0.05.

## 5. Conclusions

In this study, we successfully identified and characterized the *RBX1* gene (*Sp-RBX1*) from the transcriptome of *S. paramamosain*. The significant upregulation of *Sp-RBX1* in the ovary during stages III to V strongly suggested its crucial role in oocyte maturation. Furthermore, the distinct expression patterns observed in the hepatopancreas indicated potential additional functions in metabolic processes related to energy storage for vitellogenesis. The differential regulatory effects of FSH and E2 on *Sp-RBX1* expression highlighted a complex hormonal regulatory mechanism, likely tied to its role in the ubiquitin-proteasome pathway governing oocyte maturation. In a word, these findings provided foundational insights into the molecular mechanisms regulating ovarian development in crustaceans and proposed *Sp-RBX1* as a valuable candidate for further research into reproductive endocrinology and maturation in *S. paramamosain*.

## Figures and Tables

**Table 1:** Information on primer sequences.

Primer Type	Primer Name	Primer Sequence (5′ → 3′)
PCR primer	*Sp-RBX1*-F *Sp-RBX1*-R	GGACATCGTGGTGGACAACT TTTTGGAACTCCCACTCCCG
RT-qPCR primer	RT-qPCR-*RBX1*-F RT-qPCR-*RBX1*-R	AGGAAGAAGTTGAGGTGCCG AGTTGTCCACCACGATGTCC
18S rRNA primer	18S rRNA -F 18S rRNA -R	GGGGTTTGCAATTGTCTCCC GGTGTGTACAAAGGGCAGGG

**Table 2:** Species names and accession numbers used in this study.

Protein Name	Species	Accession Number
RBX1A	*Portunus trituberculatus*	XP_045119591.1
RBX1A	*Eriocheir sinensis*	XP_050735003.1
RBX1A	*Procambarus clarkii*	XP_045596002.1
RBX1A	*Cherax quadricarinatus*	XP_053636519.1
RBX1A	*Penaeus monodon*	XP_037796005.1
RBX1A	*Homarus americanus*	XP_042216487.1
RBX1A	*Penaeus vannamei*	XP_027226532.1
RBX1A	*Armadillidium nasatum*	KAB7497043.1
RBX1A	*Daphnia carinata*	XP_057366930.1
RBX1A	*Halyomorpha halys*	XP_014278302.1
RBX1A	*Onthophagus taurus*	XP_022905780.1
RBX1A	*Photinus pyralis*	XP_031340532.1
RBX1A	*Danaus plexippus*	XP_032511787.1
RBX1A	*Pieris napi*	XP_047522172.1
RBX1	*Betta splendens*	XM_029158827.3
RBX1	*Danio rerio*	NM_001083544.1
RBX1	*Erpetoichthys calabaricus*	XM_028815173.2
RBX1	*Mus musculus*	AAD29716.1
RBX1	*Anser cygnoides*	XP_047925861.1
RBX1	*Homo sapiens*	BAG38078.1

## Data Availability

The original contributions presented in this study are included in the article. Further inquiries can be directed to the corresponding author(s).
